# Reduction of Signal Drift in a Wavelength Modulation Spectroscopy-Based Methane Flux Sensor

**DOI:** 10.3390/s22166139

**Published:** 2022-08-17

**Authors:** Scott P. Seymour, Simon A. Festa-Bianchet, David R. Tyner, Matthew R. Johnson

**Affiliations:** Energy & Emissions Research Laboratory, Department of Mechanical and Aerospace Engineering, Carleton University, Ottawa, ON K1S 5B6, Canada

**Keywords:** methane, emission spectroscopy, mass flow, venting, oil and gas sector, measurement drift, wavelength modulation

## Abstract

Accurately quantifying unsteady methane venting from key oil and gas sector sources such as storage tanks and well casing vents is a critical challenge. Recently, we presented an optical sensor to meet this need that combines volume fraction and Doppler shift measurements using wavelength modulation spectroscopy with 2*f* harmonic detection to quantify mass flux of methane through a vent line. This paper extends the previous effort through a methodical component-by-component investigation of potential sources of thermally-induced measurement drift to guide the design of an updated sensor. Test data were analyzed using an innovative signal processing technique that permitted quantification of background wavelength modulation spectroscopy signal drift linked to specific components, and the results were successfully used to design a drift-resistant sensor. In the updated sensor, background signal strength was reduced, and stability improved, such that the empirical methane-fraction dependent velocity correction necessary in the original sensor was no longer required. The revised sensor improves previously reported measurement uncertainties on flow velocity from 0.15 to 0.10 m/s, while markedly reducing thermally-induced velocity drift from 0.44 m/s/K to 0.015 m/s/K. In the most general and challenging application, where both flow velocity and methane fraction are independently varying, the updated design reduces the methane mass flow rate uncertainty by more than a factor of six, from ±2.55 kg/h to ±0.40 kg/h. This new design also maintains the intrinsic safety of the original sensor and is ideally suited for unsteady methane vent measurements within hazardous locations typical of oil and gas facilities.

## 1. Introduction

Methane (CH_4_) is a potent greenhouse gas with a global warming potential many times stronger than carbon dioxide (~83/30 times greater over a 20/100 year time horizon when emitted from fossil fuel sources) while also having a much shorter effective lifetime in the atmosphere (~12 years vs. hundreds of years for CO_2_) [1,2]. This combination means reductions in methane emission have critical immediate term benefits for slowing global temperature rise [3]. The oil and gas sector is a critical source of methane emissions and is a key focus of global methane mitigation efforts [4,5,6]. However, field measurements using a range of techniques suggest that methane emissions from the upstream oil and gas (UOG) industry are consistently higher than reported in inventories [7,8,9,10,11,12,13], and sources such as well casing gas vents and production storage tank vents may be among the most significant contributors to unreported emissions [10,14,15,16].

Part of the difficulty in accurately quantifying methane emissions from storage tanks and well casing vents has been the lack of suitable direct measurement technologies. Specific challenges include unsteady vent rates where both flow rate and methane volume fraction may vary independently; the need for large dynamic flow range, coupled with the prerequisite of near-zero backpressure to avoid affecting the emissions process (especially important for tank sources); and the stringent requirement for equipment to meet safety standards for use in hazardous locations (i.e., Class 1, Zone 0/1 explosive gas environments). Recently, Festa-Bianchet et al. [17] proposed an optical sensor capable of satisfying these requirements and providing time-resolved, point-source methane vent rate data. The system uses wavelength modulation spectroscopy (WMS), a well-known tunable diode laser absorption spectroscopy (TDLAS) technique capable of measuring volume fraction [18,19,20] and bulk flow velocity [21,22,23,24,25,26,27,28,29,30,31] based on laser absorption by a target species in an intrinsically safe design (Class 1, Zone 0/Div. 1 in Canada/the United States) that permits the system to be safely deployed in explosive atmospheres [32,33]. In controlled tests at fixed methane fractions between 25–100%, a methane flow rate uncertainty of ±0.85 kg/h was achieved. However, under varying composition scenarios, a velocity offset error was observed that depended on methane volume fraction and required implementation of a separate empirical calibration. If the methane volume fraction varied along with flow velocity, this offset correction increased the uncertainty to ±2.1 kg/h. Moreover, continued development of the system suggested velocity measurements are subject to thermally induced drift that would affect performance in varying temperature environments. 

The goal of this study was to design and test an improved methane flux sensor, leveraging results of a comprehensive investigation of the sources of measurement drift. A novel experimental approach was used that directly measured the influence of individual components on changes in the background WMS signals that drive measurement drift. Compared to attempting to infer the effects of individual components from observed variability in the final measured methane volume fraction and/or velocity using the complete sensor, this method improved precision of the analysis, simplified the required experimental apparatus, and significantly accelerated testing. Ultimately this allowed a much broader range of components to be investigated and led to the design of a significantly improved methane flux sensor. More generally, this approach should be broadly useful in guiding design of other WMS sensors, regardless of the target gas and application. Experiments were performed to quantify measurement uncertainties and thermal stability of the new system and document improved performance over the initial system reported by Festa-Bianchet et al. [17]. The revised sensor is well-suited for field deployments to measure unsteady methane venting from oil and gas sector point sources within hazardous location flammable gas environments. 

## 2. Materials and Methods

### 2.1. Test Apparatus

**Figure 1:**
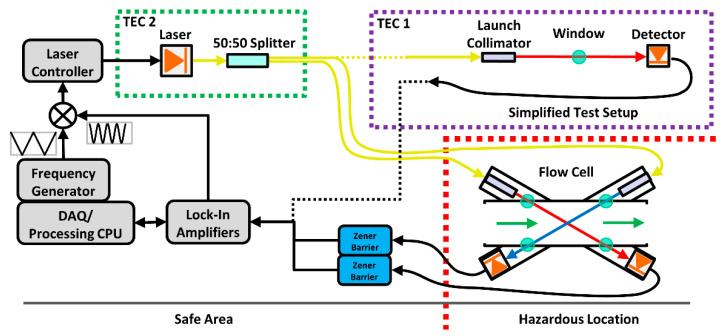
Schematic of the methane flow sensor and the simplified setup (outlined with the purple dotted line, TEC1) used during the thermal testing of key optical components. The green dotted outline (TEC 2) indicates the laser/splitter sub-assembly which was tested as a unit.

Figure 1 shows a schematic of the methane flux sensor from Festa-Bianchet et al. [17] as well as the simplified test setup used in this work to conduct thermal stability tests of specific optical components. Both systems used a distributed feedback diode laser (Eblana, EP1662-3-DM-B06-FA, Dublin, Ireland) controlled by a Stanford Research Systems laser diode controller (SRS, LDC501, Sunnyvale, CA, USA) and tuned to a center frequency of 6026.23 cm^−1^ (1659.41 nm) to detect the R 1 F1(1) absorption line of methane. A modulation frequency of 10 kHz with a depth of 0.1754 cm^−1^ (provided by a lock-in amplifier, Zurich Instruments, MFLI, Zurich, Switzerland) was superimposed on a 30 Hz triangle waveform which swept the laser’s frequency by 0.756 cm^−1^ (provided by a frequency generator, National Instruments, NI; PXI 5406, Austin, TX, USA) across the absorption transition line center. The modulation depth, corresponding to a modulation index *m* of 2.26 (where *m* is the ratio of modulation depth to the absorption line’s half width at half maximum), was selected to maximize the 2*f* response.

As in the original methane flux sensor, the laser output was coupled into single mode fiber (9/125 μm) and split equally into two beams using a fiber coupler/splitter (Thorlabs, TW1550R5A1, Newton, NJ, USA). All fiber connections used angled-physical contact (APC) connectors to minimize back-reflections. The simplified test setup differs from the original sensor by making use of only one of the splitter output fibers. This simplification was critical in reducing the number of components involved in temperature sensitivity tests, further discussed in Section 2.2.1. The single splitter output was connected to a collimator (various models) which directly irradiated a photodiode at a distance of approximately 20 cm. This spacing matched the free space distance of the original flow cell shown in the lower right of the figure. The components tested using the simplified setup were placed within a custom thermal enclosure (TEC1, see Section 2.3) which allowed the temperature to be adjusted. Similarly, for testing of the thermal stability of the laser mount and splitter, a second thermal enclosure (TEC2) was used. The photodiode signal was demodulated by the lock-in amplifier, and the harmonic outputs from the lock-in were processed in a LabVIEW environment on a host computer (NI, PXIe 8880, Austin, TX, USA). 

### 2.2. Optical Measurement Noise and Drift

Although the WMS technique has been successfully deployed in myriad applications, performance can ultimately be limited by background WMS signals (i.e., WMS signals unrelated to the absorbing gas [34]), which are most often caused by laser speckle, etalons, and varying component transmissivity [35,36,37,38]; laser output drift [39,40]; and thermal effects of detectors [37,41,42]. These spectroscopic background signals can induce non-zero offsets in measured velocity and species volume fraction. Moreover, any time-variation in these signals can manifest as measurement drift. The methane volume fraction-dependent velocity error reported in Festa-Bianchet et al. [17] was attributed to such background signals in the 2*f* harmonic. Although various strategies for mitigating the impacts of background 2*f* noise have been investigated in the literature, including background subtraction using reference laser lines [43] or inert gas purging [44]; mechanical jitter to average out background signals [20,37]; thermal-stabilization [20]; and filtering of sinusoidal WMS signals [45], the primary strategy is always to avoid or minimize these signals as much as possible through judicious optical design and component selection. Moreover, most of these mitigation strategies are not easily applied to a field-deployable instrument. 

#### 2.2.1. Quantification of Background WMS Signals

Initial efforts to quantify signal drift of the methane flow sensor focused on changes in the measured outputs of the complete optical system (i.e., volume fraction and velocity) in different configurations. However, the variable nature of signal drift and the number of required components in the fully assembled, dual-beam system made it difficult to reliably attribute changes in performance back to individual components. Moreover, longer duration stability tests required the light-absorbing gas be maintained at a stable volume fraction and flow rate. This proved difficult to achieve especially at velocities near zero where even minor temperature differences could induce convection currents on the order of 0.1 m/s.

Instead, we present a novel, simple method for quantifying changes in WMS background signals that drive overall measurement offsets and drift. This method involves recording a reference background signal (i.e., with no absorbing gas) at the beginning of the experiment, and subsequently monitoring any changes to this signal while an external disturbance, in this case a temperature change, is imposed on a subset of the system. Importantly, this approach only requires monitoring the signal from a single laser beam and it removes the need for any light-absorbing gas. This greatly simplifies the experimental setup and accelerates testing, allowing a broader range of components to be investigated as part of creating an optimized system design.

A 30-s average of a given harmonic signal was used as the reference background ($WMS_{ref}$) against which changes could be monitored. Changes to the background WMS signals of a test configuration ($WMS_{test}$) were evaluated based on the difference between the test and reference background signals. The absolute value of this difference, integrated across each point i of the laser sweep (IBD; integrated background difference), was computed as a convenient single-value parameter to characterize the change in background signals between two configurations.

(1) $$IBD=\overset{N}{\underset{i=1}{\sum }}\left|WMS_{test}\left(i\right)-WMS_{ref}\left(i\right)\right|$$

**Figure 2:**
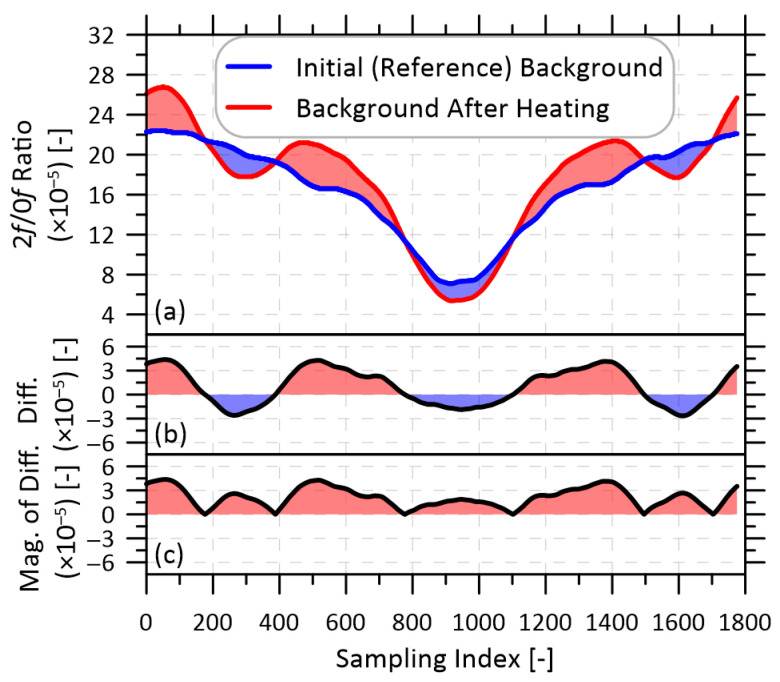
Illustration of a change in background signal of a device under thermal test. (**a**) The 2*f*/0*f* background signals before (reference) and after the device was heated; (**b**) The difference between the backgrounds; and (**c**) the magnitude of this difference. In this example, the integrated magnitude of the background difference across the scan is IBD = 0.0377 [-].

Changes in IBD values among different selections and configurations of optical components were used to guide the design of a system that maximized measurement accuracy and minimized temperature-induced drift. Figure 2a shows an example of the difference between the reference 2*f*/0*f* background and the background signal after heating the components under test by 12 °C; the difference between the background signals (b) and magnitude of this difference (c) are also shown. 

### 2.3. Thermal Enclosure and Component Testing Program

**Figure 3:**
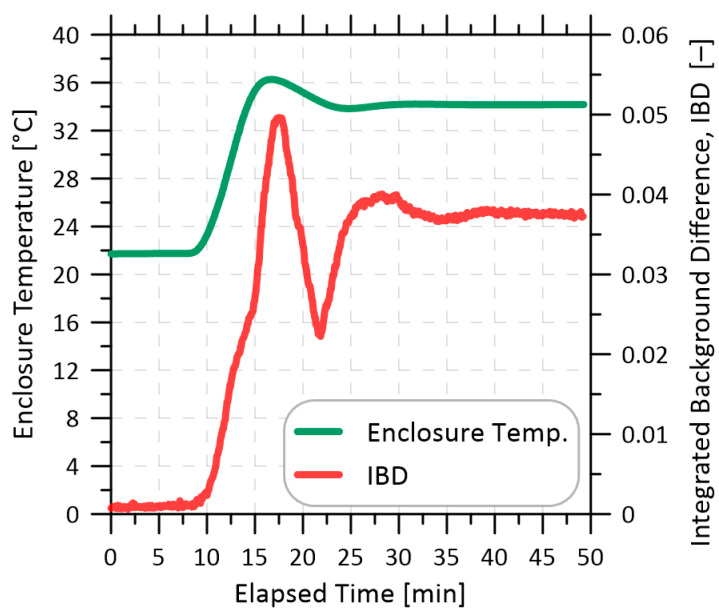
Typical progression of measured enclosure temperature and integrated background difference (IBD).

A custom-built thermal enclosure was used to test the temperature sensitivity of individual measurement-system components and their influence on WMS background signals as quantified via the IBD. The enclosure had an internal volume of 0.009 m^3^ (30 cm × 30 cm × 10 cm) and the air temperature within was controlled via a thermoelectric heater-cooler (TEC) unit (TE Technology, CP-121HT, Traverse City, MI, USA). Component(s) under test were placed within the enclosure, which was heated from 22 °C to 35 °C while the IBD was measured as illustrated in Figure 3. The initial WMS background signal at 22 °C was monitored for at least 7 min to ensure the WMS signal was stable and a 30-s average was recorded as $WMS_{ref}$. The internal temperature of the enclosure was then increased to 35 °C over a period of 10 min and maintained for at least 35 min, after which a new 30-s averaged WMS signal was recorded ($WMS_{test}$) and the IBD was calculated via Equation (1). For all component-test results presented in this work, this procedure was repeated three times. After each test, the components under test were allowed to return to 22 °C and all optical elements were reconnected and realigned to ensure that results were not specific to a particular alignment. 

## 3. Results

### 3.1. Optical Component Testing

**Figure 4:**
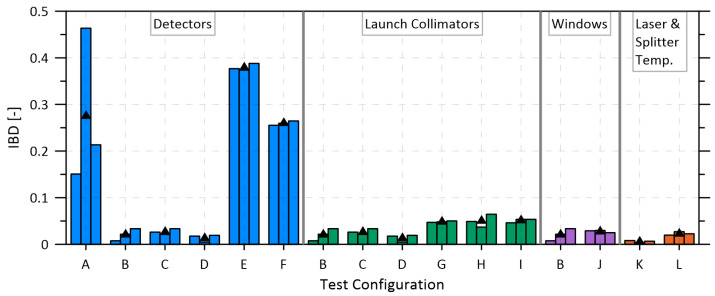
Integrated background difference (IBD) results for component tests from Table 1 under heating cycles, repeated three times per configuration with corresponding average values plotted as black triangles.

As summarized in Table 1 and Table 2, initial experiments investigated the four main components in the optical measurement path: the laser splitter (and associated connectors), launch collimator, optical windows, and photodiode detector. A summary of the test configurations is provided in Table 1 and details of the individual tested components are shown in Table 2. Comparison results, evaluated in terms of the IBD, are plotted in Figure 4.

By far the largest changes in IBD from heating, and the greatest differences among different options, were observed in the photodetector tests. Although Indium Gallium Arsenide (InGaAs) detectors are well-suited to near-infrared spectroscopic applications due to their linear response (within 0.08% uncertainty [46]) and ability to remain linear over a large power band when reverse biased [47], they can be affected by temperature near their band edges [37]. Experiments by Woodward et al. [37] showed that while the responsivity of InGaAs photodetectors is unaffected by temperature in the middle of their range (~1000–1600 nm), changes in responsivity of up to 3%/°C were measured near the band edges at 1700 nm. Interestingly, one of the worst performing photodetectors in Figure 4 is the DET1 diode used by Festa-Bianchet et al. [17], which had a manufacturer specified cut-off of 1700 nm. Interpolating the data of Woodward et al. [37] suggests that at the present measurement wavelength of 1660 nm, a change of ~0.4% per degree might be expected which may have contributed to signal drift and WMS background changes in the system of Festa-Bianchet et al. [17]. 

However, test results were similarly poor with an extended range InGaAs photodetector (ext-InGaAs, band edge near 1870 nm). Although initially unexpected, this result is likely due to the less uniform response of photodiodes with extended range characteristics. InGaAs photodiode spectral bands are extended to longer wavelengths by changing the ratio of Indium and Gallium in the semiconductor matrix (i.e., In_x_Ga_1−x_As); in this way, responsivity band edges can be extended to 2.5 μm [48,49,50]. Despite the shift of band edge away from the working wavelength, extended-wavelength photodiodes have also been shown to exhibit higher surface response non-uniformity and non-linearity [37,48]. This increased non-uniformity is caused by the mismatched lattice structure of the ext-InGaAs substrate which causes material strain and defects. Although improvements have been reported in ext-InGaAs manufacturing to reduce such effects [51] and small reverse biasing has been shown to improve surface uniformity [52], it was not clear from the literature how these effects would combine. 

The tests in Figure 4 show that the ext-InGaAs photodetectors performed poorly (i.e., IBD was large) whether thermal stabilization was enabled (DET5-C, test configuration E) or disabled (DET5-UC, test F). The larger IBD with active diode cooling (E) is assumed to be caused by an increase in temperature swings of the photodetector case and/or its built-in window. The TEC within the photodetector moves thermal energy to the backside of the photodiode to maintain its surface temperature. However, during heating, this photodiode stabilization would introduce temperature differences between the diode surface and its case (TO-66 semiconductor package), which could affect the transmissivity or speckle pattern at the photodetector window before the beam interacts with the non-uniform surface of the photodiode [53].

The remaining three detectors (DET2, DET3, DET4) exhibited substantially lower background change when subject to the same temperature cycling. Although DET4 had the lowest mean IBD during temperature cycling ($IBD_{DET4}=$ 0.014), slightly below that of DET2 ($IBD_{DET2}=$ 0.021), the difference was not statistically significant (p= 0.47). Moreover, use of an amplified detector (i.e., DET4) within the intended Class I, Zone 0/1 hazardous location measurement zone would require the amplification circuit to be certified as intrinsically safe. By contrast, the unamplified DET2 is considered a simple device and can be safely deployed by inserting a readily available low-resistance Zener barrier in the current loop (e.g., Pepperl + Fuchs, 937ZH-DPBN-2, Mannheim, Germany). Therefore, the unamplified DET2 photodetector (Thorlabs, SM05PD5A, Newton, NJ, USA) was selected to replace the detectors used in Festa-Bianchet et al. [17], resulting in an order of magnitude reduction in WMS background change due to the photodiode.

Although the results of Figure 4 show that the choice of launch collimator was of secondary importance, test configurations using the reflective collimators (tests B–D) had consistently lower IBD than configurations using the singlet collimators of Festa-Bianchet et al. [17]. This difference is attributed to variation in the shape and/or position of the beam striking the detector which can introduce changes to the WMS background signal due to a non-uniform photodetector surface [53]. It should be noted that mounts for the singlet and reflective collimators were also necessarily different. Some of the observed reduction in IBD may be attributable to the Polaris-K05S1 mount used with the reflective collimator, which was specifically designed to minimize deflection due to temperature changes [54]; this same mount was not directly compatible with the singlet collimator. Nevertheless, compared to the original COL2 singlet collimator assembly used by Festa-Bianchet et al. [17], the presently selected COL1 assembly produced a statistically significant reduction (*p* < 0.00001) in the IBD from 0.05 to 0.02 across all tests with all detectors. 

In addition to collimators and photodiodes, windows are necessary in the flow cell to contain the measured gas and to separate it from the optics. Fortunately, test configurations B and J revealed no statistically significant change (*p* = 0.41) in IBD from inserting an angled wedged window into the optical path. Finally, results from test configuration L, which investigated heating of the laser diode assembly including the DFB laser diode, the 50:50 fiber-based splitter, and associated fiber connections, showed a mean IBD of 0.019 [-] over a 12 °C change. This IBD is similar in magnitude to that of the selected reflective collimator assembly (DET2, test configuration B) and was a statistically significant increase (*p* < 0.006) over a reference case (K) in which TEC2 was used to hold the temperature of these components constant. Direct heating of the individual components within the laser diode assembly using a heat gun suggested that it was specifically the FC/APC single mode fiber connections that contributed to measurement drift. Drift magnitudes were seen to increase substantially if the fiber connectors had not been properly cleaned prior to connection. All connectors were inspected by microscope and cleaned with a fiber cleaning cloth prior to final assembly. More importantly, for the final system design presented below, the laser driving components and splitter (specifically including the FC/APC connections) were temperature stabilized in a thermal enclosure (TEC2) as recommended by Schoonbaert et al. [20]. 

### 3.2. Detection Harmonic Selection

The preceding tests were all completed using 2*f*/0*f* detection. Although the 2*f* harmonic is the most common choice for WMS [35], the optimal harmonic is hardware-dependent and in some cases other harmonics (specifically 4*f* or 6*f*) can give greater signal-to-background ratios despite the decreased signal strength of the harmonics themselves [55]. It is also common to normalize the detection harmonic by another harmonic to remove the dependence on absolute light intensity [31,56,57]. So far in this work the 2*f* was divided by a linear interpolated 0*f* (i.e., a harmonic signal demodulated at 0 Hz to preserve only DC terms [17]). The interpolation minimizes the intensity loss due to methane absorption and is an effective estimate of the absorption free intensity, which compensates for any change in bulk transmissivity through the flow cell. However, several studies have found success using the 2*f*/1*f* harmonic ratio specifically for velocity measurements [31,38,58,59] rather than the 2*f*/0*f*. To investigate the optimal choice of detection harmonic for the present system, experiments were performed to compare the performance of six possible combinations of the 2*f* and 4*f* signals normalized by either the raw 0*f* signal, a linearly interpolated 0*f* (“0*f* (int.)”) signal as in [17] (i.e., where the 0*f* is linearly interpolated across the region of the absorption peak), or the 1*f* signal. Combinations using the 6*f* harmonic were not considered following preliminary experiments that found they had lower signal-to-background ratios than corresponding normalizations of the 4*f*. 

**Figure 5:**
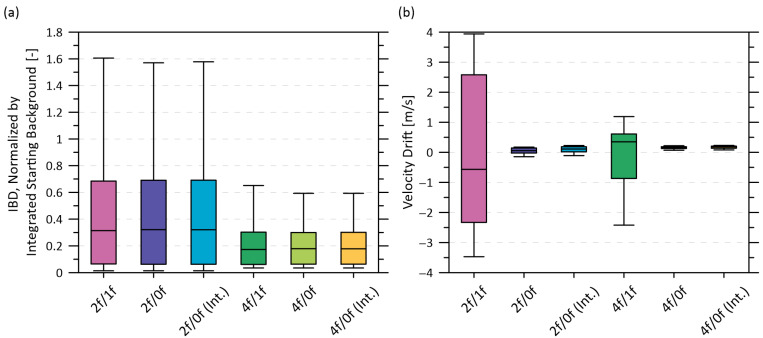
(**a**) Comparison of integrated background difference (IBD) normalized by the integrated background at the start of the heating test when using different WMS harmonics. (**b**) Comparison of harmonic ratio performance in terms of velocity drift using the complete flow cell.

Figure 5a shows box and whisker plots of IBD for each WMS harmonic, as measured during the combined experiments A–F presented in Section 3.1 using the six different detectors of Table 2. The lock-in amplifier was programmed to log four concurrent harmonics for each test: 0*f*, 1*f*, 2*f*, and 4*f*. To permit comparison given the differences in magnitude among the harmonics at the same methane absorbance, the IBD is normalized by the integrated background ($WMS_{ref}$) for each harmonic prior to the start of the heating cycle. From these tests, it appears that all 4*f* harmonic combinations experience roughly a factor of two less background change than the 2*f* harmonics during the heating tests. However, changes in the background signal level alone do not determine the final amount of measurement drift, particularly for velocity. The different magnitudes of the harmonics affect relative signal to noise ratios and, in particular, the different shapes of the harmonics yield different sensitivities to background noise when the up- and downstream laser signals (each with independent background optical noise characteristics) are cross-correlated to measure velocity. 

To investigate this further, additional experiments were performed using the complete flow cell described in Section 3.3.1, to measure velocity drift as the entire flow cell was subjected to controlled heating (see Section 4.1). As plotted in Figure 5b, it appears that the sensitivity of the 4*f* to background changes offsets any benefit of the smaller IBD in Figure 5a such that measured velocity drift under temperature cycling is similar to the 2*f* harmonics. Moreover, these results show that the drift with 1*f*-normalized harmonics is worse than with either of the 0*f*-normalized harmonics. This was originally unexpected since previous studies [31,58] have suggested that 1*f*-normalized harmonics are well-suited to sensitive Doppler shift detection due to their the sharply rising shape (driven by the 1*f* falling toward zero at peak absorption). However, this same sharply rising 1*f*-normalized features is potentially more sensitive to background WMS signals at that same wavelength, especially when these backgrounds differ between the upstream and downstream laser measurement lines. The present results suggest that this sensitivity makes the 1*f*-normalized harmonics less stable than other harmonic combinations when the cell is subjected to thermal cycling. 

Based on these results, the 2*f*/0*f* was retained as the detection signal. Although the measured velocity drift of the 4*f*/0*f* was slightly lower than that of the 2*f*/0*f* (0.15 ± 0.06 vs. 0.04 ± 0.1 m/s), the latter had superior instantaneous velocity precision (standard deviation of 0.02 m/s for 2*f*/0*f* versus 0.04 m/s for 4*f*/0*f*, both at 50% methane volume fraction). The choice of normalizing the 2*f* signal by either the interpolated 0*f* or the raw 0*f* signal did not have a measurable impact on velocity drift. 

### 3.3. Improved Methane Flux Sensor

#### 3.3.1. Updated Methane Sensor Design

**Figure 6:**
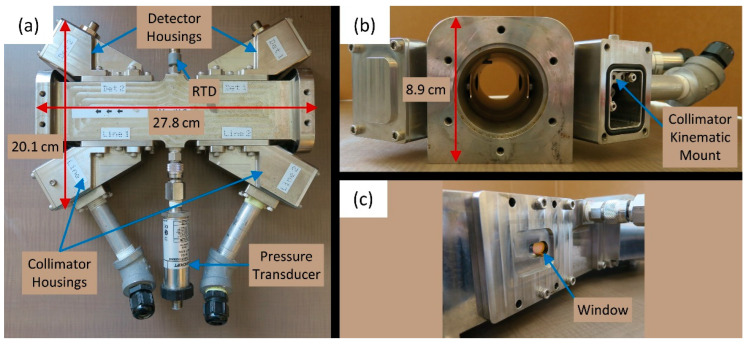
Updated flow cell. (**a**) Top view showing the pipe nipples and cable glands (bottom of the figure) that are used to protect and seal the fiber optic cables. (**b**) Side view showing the clear bore of the flow cell, with the RTD probe visible in the upper left, along with the minimal intrusion of the windows that is key to achieving negligible pressure drop. (**c**) Close-up of fused silica windows.

The methane flow system presented in our previous work [17] was redesigned based on the preceding results. The new design used reflective collimators in Polaris mounts (COL1), unamplified photodiodes (DET2), wedged windows (WW), and stabilized the temperature of the laser driving system. Additionally, a new flow cell was constructed from Invar (an iron-nickel alloy with a low coefficient of thermal expansion, *CTE*_Invar_ = ~1.2 ppm/°C) that could be compared with the original aluminum flow cell (*CTE*_Alu_ = 21–24 ppm/°C). To closely match the thermal expansion of the new sensor head, the wedged windows (WW40530, Thorlabs, Newton, NJ, USA) were made of fused silica (0.55 ppm/°C) instead of N-BK7 (7.1 ppm/°C) as used in the original sensor, with a custom anti-reflective coating designed to minimize reflectance at the beam’s incidence angle. As in Festa-Bianchet et al. [17], the photodiode circuits included Zener barriers (Pepperl + Fuchs, 937ZH-DPBN-2, Mannheim, Germany) to conform to hazardous location safety requirements. A pre-computed look-up table (LUT) of coefficients was generated for the methane absorption line at 6026.23 cm^−^^1^ (1659.1 nm) using spectroscopic data from HITRAN 2012 and a Lorentzian lineshape [60]. This LUT was used to calculate theoretical peak heights of the second harmonic (2*f*) across a range of temperatures (−30 to +70 °C, steps of 1 °C), pressures (90 to 105 kPa, steps of 2.5 kPa), and methane volume fractions (0 to 100%, steps of 1%). The calculated theoretical 2*f* peak heights, scaled by the estimated absorption-free intensity, were then linearly interpolated using the measured 2*f* peak height, gas temperature, and pressure to obtain a theoretical methane volume fraction. This methane volume fraction was then combined with intrinsically-safe measurements of temperature and pressure to calculate gas density, which was combined with the measured flow velocity to yield the methane mass flow rate. The updated flow cell is shown in Figure 6, along with its principal dimensions and components.

## 4. Discussion

### 4.1. Temperature Stability of the Updated Sensor

**Figure 7:**
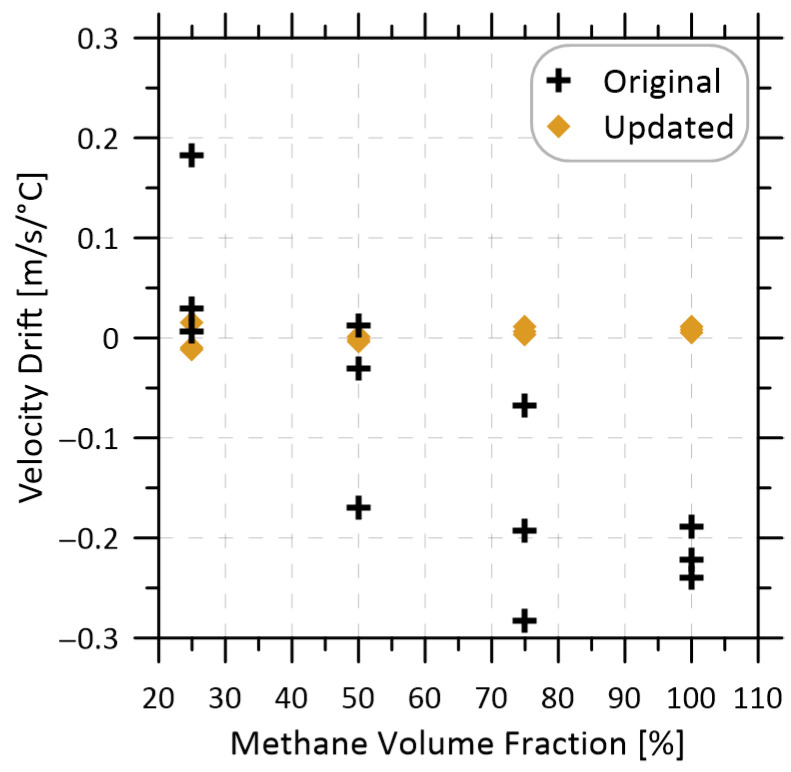
Measured velocity drift at different methane volume fractions during controlled heating of the original and updated flow cells.

To validate the combined improvements made to the system, two insulated enclosures large enough to contain either the original methane sensor (Original) or the new methane sensor (Updated) were constructed. Two fans and a 90 W heater within the enclosure (operated using a PID controller) raised the temperature from ambient to 37 °C (roughly a 12 °C change) over the course of 1.5 h to ensure that a stable temperature had been reached. Valves were installed on both ends of the two flow cells to seal various methane-nitrogen mixtures within the cells, such that any drift in the measurements due to temperature change could be monitored at stable methane volume fractions. Figure 7 plots the measured velocity drifts during these tests, which have been normalized by the measured temperature change to permit direct comparison.

The mean velocity drift of the updated flow cell was approximately 25 times lower than that of the original. Across three repeats at 25, 50, 75, and 100% methane, the original system had a mean drift of −0.1 ± 0.33 m/s/°C while the revised sensor had a mean drift of 0.004 ± 0.021 m/s/°C, both at 95% confidence. Interestingly, the original sensor showed an apparent negative drift correlation with methane fraction (R^2^ = 0.67), which may have been caused by changes to the background WMS signals [17]. By contrast, the updated sensor shows no apparent correlation of drift with methane fraction (R^2^ = 0.24) as further discussed below. 

**Figure 8:**
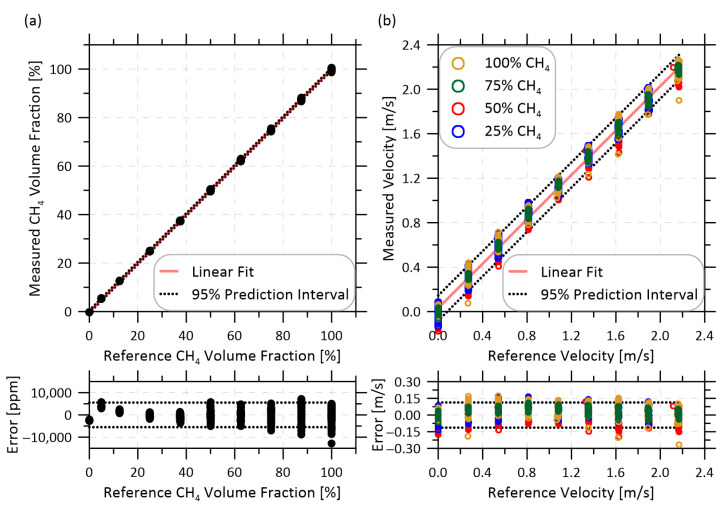
(**a**) Calibrated methane volume fraction and (**b**) bulk velocity measurements at four methane volume fractions of the updated sensor, including overall prediction intervals at 95% confidence. Corresponding measurement error is shown in the respective lower plots.

Although reducing the impact of background signal drift on the measured velocity was the primary focus of this study, the impact on the methane volume fraction measurement was also estimated. Since the magnitude of drift in the measured methane volume fraction was too low to be detected beyond the 0.5% precision of the instrument (see Figure 8a), the drift was instead inferred from analysis of measured changes in the background WMS signals. With each methane sensor purged with nitrogen, the background change at the 2*f* peak location was measured during heating of the measurement cell. The magnitude of this change was then used to calculate the maximum expected change in measured methane volume fraction, which would occur at 100% methane. Results from three repeated experiments show that the absolute methane volume fraction can be expected to vary by no more than 0.02% ± 0.06%/°C for the original system versus 0.007% ± 0.009%/°C for the updated design, both at 95% confidence. 

### 4.2. Measurement Uncertainty of the Updated Sensor

Performance testing of the updated system was completed to quantify final measurement uncertainties and any improvements over the original design. Bottled compressed methane (ME 2.0-T, 99.0% CH_4_, Linde Canada, Mississauga, ON, Canada) and nitrogen (NI 5.0UH-T, 99.999% N_2_, Linde Canada) were metered using two calibrated thermal mass flow controllers (MFCs, EL-FLOW F-202AV-M10-RAD-55-V, Bronkhorst, Ruurlo, The Netherlands) and mixed to create a range of controlled flow rates and volume mixing ratios that were directed through the optical measurement cell. Velocity and methane fraction were recorded at a sampling rate of 1 Hz. Calibration curves for methane fraction and velocity measurements (polynomial and linear, respectively, as in Festa-Bianchet et al. [17]) were derived for the updated sensor and applied to subsequent test data. Tests spanned the full range of 0 to 100% methane at velocities of up to 2.2 m/s, limited by the maximum flow rates of the MFCs. These ranges corresponded to a methane mass flow range of 0 to 11 kg/h. Experiments were repeated five times over the course of three days to assess repeatability. Figure 8 plots the combined data from all 5 tests. The dotted lines indicate prediction intervals at 95% confidence. These results demonstrate a measurement uncertainty on methane volume fraction within ±0.5% methane and velocity within ±0.1 m/s. 

**Figure 9:**
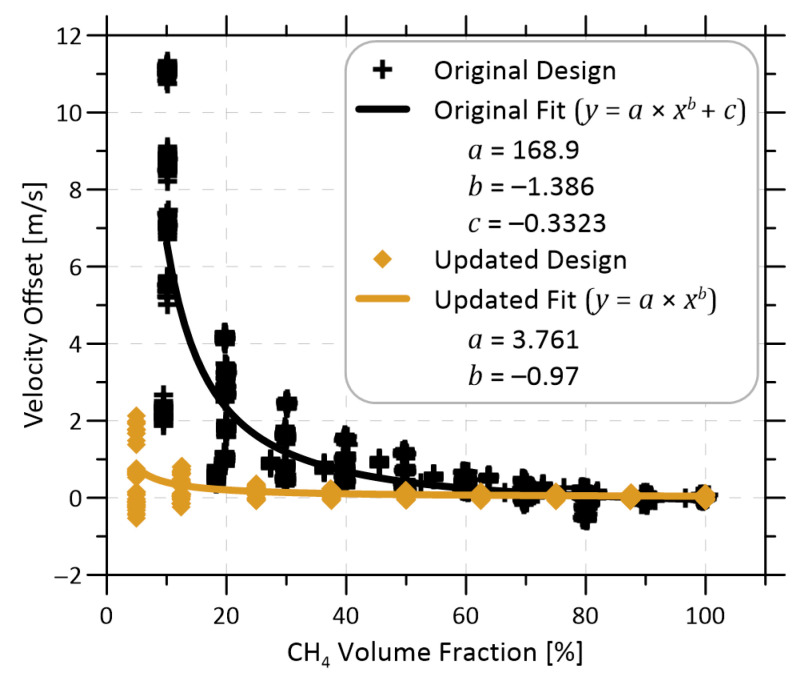
Comparison of the dependence of velocity offset on methane volume fraction between the original flow cell design used in Festa-Bianchet et al. [17] and the updated design from the current work.

As previously mentioned, the original methane mass flow sensor required an empirical velocity offset correction that was dependent on methane volume fraction. This velocity offset placed a significant limitation on the applicability of the sensor, restricting its usefulness to specific scenarios where the methane volume fraction was not expected to vary. Importantly, no such correction was applied to the data for the updated sensor shown in Figure 8. To investigate this further, Figure 9 reproduces the original velocity offset data and derived correction function from Festa-Bianchet et al. [17], and overlays equivalent test data for the updated flow cell from the present work. While in both cases, the apparent velocity offset became undefined towards zero methane fraction as there is no longer a methane signal to detect, the present data demonstrate that the offset is negligible at methane fractions ≥ 25%. This is a significant improvement over the previous design.

**Figure 10:**
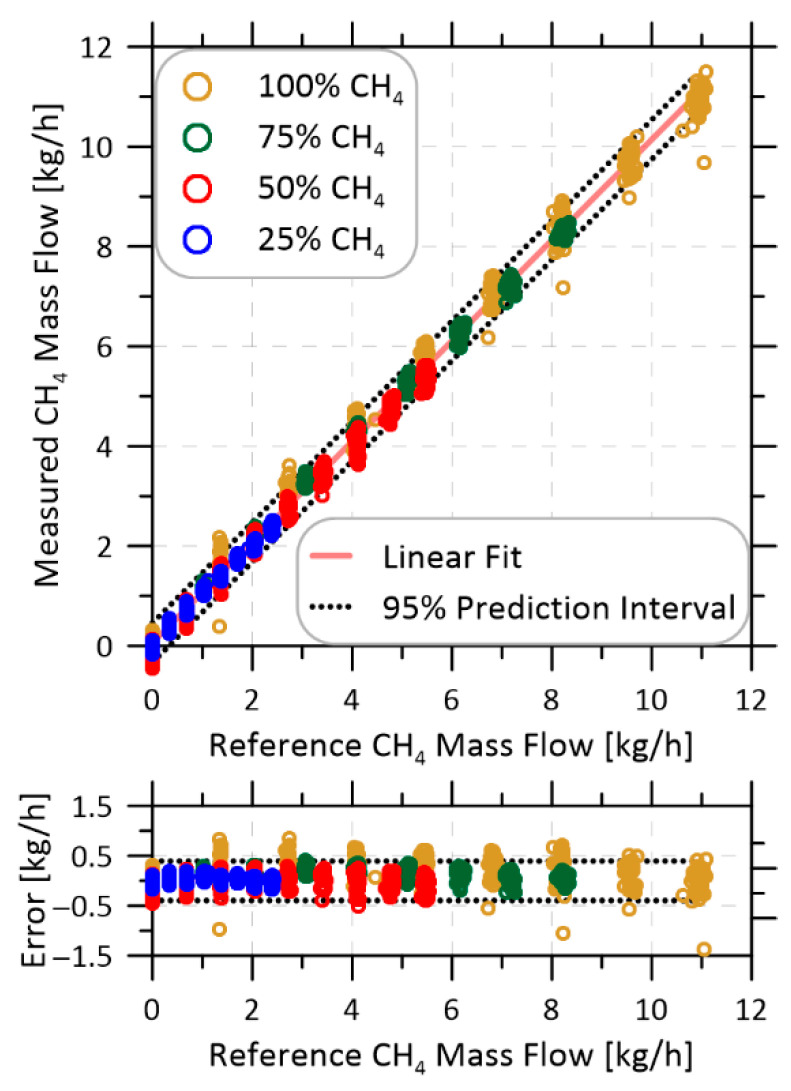
Measured methane mass flow measurements compared with set rates delivered by mass flow controllers. For methane fractions ≥ 25%, the sensor achieved a ±0.40 kg/h uncertainty at 95% confidence.

Finally, Figure 10 plots measured methane mass flows using the updated system versus prescribed flow rates from the mass flow controllers. The different colors identify measurements made at different methane volume fractions between 25–100%. The lower plot shows the error in the measured flow rate and for both plots the dotted lines show prediction intervals at 95% confidence. Over the full range of data in Figure 10, the updated methane sensor achieved a mass flow rate uncertainty of ±0.40 kg/h at 95% confidence. This is more than a factor of six improvement over the previous sensor that reported a ±2.55 kg/h uncertainty under identical conditions, and more than a factor of two improvement over the previous best case ±0.85 kg/h uncertainty for scenarios where the methane volume fraction was stable. 

### 4.3. Outlook

Based on the preceding results, the newly designed methane flux sensor is capable of accurate, stable, time-resolved (1 Hz) quantification of methane flows, while being safely deployable in Class I, Zone 0/1 hazardous locations due to presence of flammable gases. This makes it ideally suited for applications in the upstream oil and gas industry, where there is an urgent need for technologies to quantify unsteady methane emissions from key venting-sources such as liquid storage tanks and casing gas vents that can dominate total emissions [10,11]. As a technique for quantifying tank venting, the sensor could be used to assess tank venting models [61,62,63] as well as assumed regional compositions of the vented gas [64,65]. Coupled with tank level or other measurements of produced oil volumes, the sensor could be used to measure time-resolved gas production or casing gas venting sufficient to calculate real-time gas-oil-ratios (GOR). This could overcome a key barrier to accurate methane emissions reporting, where poorly estimated GOR has been implicated in several studies as a key reason for underestimation of venting in current inventories [11,14,66,67].

## Figures and Tables

**Table 1:** Summary of Test Configurations.

Component under Test	Test Configuration	Detector	Collimator	Window	Laser, Splitter	Harmonic
Detectors	A	DET1	COL1	None	Temp.-stabilized	2*f*/0*f*
	B	DET2	COL1	None		
	C	DET3	COL1	None		
	D	DET4	COL1	None		
	E	DET5-C	COL1	None		
	F	DET5-UC	COL1	None		
Launch Collimators	B	DET2	COL1	None		
	C	DET3	COL1	None		
	D	DET4	COL1	None		
	G	DET2	COL2	None		
	H	DET3	COL2	None		
	I	DET4	COL2	None		
Windows	B	DET2	COL1	None		
	J	DET2	COL1	WW		
Laser/Splitter	K	DET2	COL1	None	Temp.-driven	
	L	DET2	COL1	None	Temp.-stabilized	

**Table 2:** Tested Optical Components.

Component Type	Component ID	Manufacturer, Model	Description
Detectors	DET1	Thorlabs, SM05PD4A	1-mm detector diameter, 800–1700 nm range, InGaAs, unamplified, 0 V reverse bias
	DET2	Thorlabs, SM05PD5A	2-mm detector diameter, 800–1700 nm range, InGaAs, unamplified, 0 V reverse bias
	DET3	Thorlabs, PDA10CS	1-mm detector diameter, 800–1700 nm range, InGaAs, transimpedance amplified, 5 V reverse bias
	DET4	Thorlabs, PDA20CS	2-mm detector diameter, 800–1700 nm range, InGaAs, transimpedance amplified, 5 V reverse bias
	DET5-C	Laser Components, IG19X1000S4i	1-mm detector diameter, 800–1870 nm range, extended-InGaAs, transimpedance amplified, 0 V reverse bias, TEC-stabilized
	DET5-UC	Laser Components, IG19X1000S4i	1-mm detector diameter, 800–1870 nm range, extended-InGaAs, transimpedance amplified, 0 V reverse bias, TEC-stabilized disabled
Launch Collimators	COL1	Thorlabs, RC02APC	Mirrored reflective collimator held in a 2-axis kinematic mount (Thorlabs, POLARIS-K05S1), measured 1/e^2^ beam diameter ~1.25 mm
	COL2	Thorlabs, F110APC-1550	Singlet lensed collimator with an anti-reflective coating mounted in a threaded 2-axis kinematic mount (Thorlabs, KAD12F), measured 1/e^2^ beam diameter ~1.29 mm
Windows	WW	Thorlabs, WW10530-C	3-mm thick N-BK7 window, 30 arcmin wedge angle, and anti-reflective coating (1050 to 1700 nm;). Window was positioned 4 cm from the PD, and angled at 7.4 degrees to minimize back-reflections
	NW	n/a	No window
